# An interpretable machine learning framework for opioid overdose surveillance from emergency medical services records

**DOI:** 10.1371/journal.pone.0292170

**Published:** 2024-01-30

**Authors:** S. Scott Graham, Savannah Shifflet, Maaz Amjad, Kasey Claborn

**Affiliations:** 1 Department of Rhetoric & Writing, Center for Health Communication, University of Texas at Austin, Austin, TX, United States of Amedrica; 2 Addiction Research Institute, University of Texas at Austin, Austin, TX, United States of Amedrica; 3 Steve Hicks School of Social Work, University of Texas at Austin, Austin, TX, United States of Amedrica; Jeonbuk National University, REPUBLIC OF KOREA

## Abstract

The goal of this study is to develop and validate a lightweight, interpretable machine learning (ML) classifier to identify opioid overdoses in emergency medical services (EMS) records. We conducted a comparative assessment of three feature engineering approaches designed for use with unstructured narrative data. Opioid overdose annotations were provided by two harm reduction paramedics and two supporting annotators trained to reliably match expert annotations. Candidate feature engineering techniques included term frequency-inverse document frequency (TF-IDF), a highly performant approach to concept vectorization, and a custom approach based on the count of empirically-identified keywords. Each feature set was trained using four model architectures: generalized linear model (GLM), Naïve Bayes, neural network, and Extreme Gradient Boost (XGBoost). Ensembles of trained models were also evaluated. The custom feature models were also assessed for variable importance to aid interpretation. Models trained using TF-IDF feature engineering ranged from AUROC = 0.59 (95% CI: 0.53–0.66) for the Naïve Bayes to AUROC = 0.76 (95% CI: 0.71–0.81) for the neural network. Models trained using concept vectorization features ranged from AUROC = 0.83 (95% 0.78–0.88)for the Naïve Bayes to AUROC = 0.89 (95% CI: 0.85–0.94) for the ensemble. Models trained using custom features were the most performant, with benchmarks ranging from AUROC = 0.92 (95% CI: 0.88–0.95) with the GLM to 0.93 (95% CI: 0.90–0.96) for the ensemble. The custom features model achieved positive predictive values (PPV) ranging for 80 to 100%, which represent substantial improvements over previously published EMS encounter opioid overdose classifiers. The application of this approach to county EMS data can productively inform local and targeted harm reduction initiatives.

## Introduction

The opioid crisis has reached historic levels in the US and remains a growing and urgent public health concern. Recent data from the Centers for Disease Control and Prevention (CDC) revealed that over 82,998 people in the United States died from an opioid overdose in 2022 [1]. Estimates indicate that there were 2.88 million opioid-related emergency department (ED) admissions in 2016 and 2017 [2]. More recent analyses also show a 10.5% increase in opioid-related ED visits since the onset of the COVID-19 epidemic [3]. As striking as these figures are, they are generated from data that suffer from a number of data quality, delay, and under-reporting issues [4–7]. CDC tracking of fatal opioid overdoses relies primarily on collecting cause of death data from death certificates that suffer from significant time lags and may be underreported in some states, such as Texas [5]. The latest estimates indicate that 84% of reports are complete within 13 weeks [8]. Additionally, CDC’s Drug Overdose Surveillance and Epidemiology (DOSE) System tracks non-fatal overdoses through surveillance of emergency department admissions data in 20 states and Washington, DC. Unfortunately, the reliance on ED admissions for non-fatal opioid overdose surveillance markedly undercounts overdoses among people who are stabilized by emergency medical services (EMS) and subsequently refuse transport [9] or who have no interaction with healthcare systems at all [7]. ED records surveillance systems also rely significantly on diagnostic codes from the tenth revision of the International Classification of Diseases (ICD-10). Unfortunately, ICD-10 codes have been shown to be unreliable measures of substance use and are likely to underestimate true use rates [10]. Finally, overdose death and ED admissions are delayed surveillance systems that often lack critical information that could be used to productively inform public health responses and community-centered harm reduction initiatives. The White House Office of National Drug Control Policy and community harm-reduction organizations both report that their efforts are stymied by a lack of accurate, timely, and location-aware opioid overdose data [5, 7].

In recognition of these data limitations, there have been a number of recent efforts aiming to better address the opioid epidemic through the use of machine learning (ML) applications [11–17]. These include both initiatives designed to support individual risk assessment [16, 17] and broader population-level surveillance systems [11–15, 18]. Among these population-level studies, there appears to be a growing consensus that current surveillance efforts must be augmented with additional datasets that can better support near real-time and location-aware monitoring [5, 7, 13, 14, 19]. For example, two studies explore the potential benefits of enhancing current surveillance efforts with analyses of web searchers and social media chatters about opioid use [13, 19]. Additionally, the use of EMS encounter data appears to be an especially promising avenue for future development [14, 20]. EMS are typically involved in the full range of opioid overdose events. They respond to overdose deaths, transport to EDs, and stabilize in the field for patients who refuse transport. While no single system will account for all opioid overdoses, EMS records have the potential to be more encompassing than either death certificates or ED admissions data [21].

However, the use of EMS records is not without its challenges. Although there are clear limitations to the use of ICD-10 codes, they do provide a specific diagnosis that is relatively easy to track. EMS records typically include two data fields that could be considered analogous to diagnoses. These are call nature and on-scene impressions. Call natures are the dispatchers’ best guess as to the nature of the condition based on caller description. As such, they are a highly unreliable proxy for diagnosis. While emergency medical technicians (EMTs) and paramedics do not assign ICD-10 codes, they frequently register impressions that can be similar to diagnoses, but are often assessments of presentation, e.g., “altered mental status.” Additionally, given the frequently active nature of EMS encounters, initial impressions are often revised in the field. All-in-all, this creates a situation where simple data queries will be insufficient to identify overdoses. One primary approach to addressing this issue has been to link EMS records to ED admissions so that ICD-10 codes can be used for ML training and validation [14]. However, while this technique solves one problem, it removes the most vulnerable populations from available training data. That is, records for patients who deny transport subsequent to treatment in the field cannot be included in the dataset because they do not have a corresponding ED record and diagnostic code. Fortunately, recent advances in text analysis and ML applications have the potential to identify opioid overdoses from EMS narrative encounter data without relying on diagnoses subsequent to ED admission.

This study reports on our development of an interpretable and computationally inexpensive opioid overdose classification model that can be used to support near real-time, location-aware monitoring for use in public health surveillance and harm reduction resource allocation. We used a rigorous, expert-indexed content analysis approach to identify opioid overdoses in EMS encounter data from three Texas counties. We subsequently developed an array of models using three competing feature engineering frameworks: term frequency-inverse document frequency (TF-IDF), clinical concept vectorization with Cui2Vec [22], and a custom “flags” approach. We compared the performance of these candidate frameworks using standard ML metrics. Our results indicate that the custom flags approach can meet or exceed the performance of both TF-IDF and more advanced vectorization approaches while being computationally less expensive. Finally, we present an analysis of variable importance for the lightweight model to demonstrate the ease of interpretation.

## Methods

### Data and sources

The data for this study came from three Texas county EMS providers, those for Travis, Williamson, and El Paso Counties. These counties were selected to represent distinct Texas county profiles. Travis County is predominantly urban and White, with 33.6% of residents identifying as Hispanic or Latino in the 2020 census. Travis County has a total population of 1,250,844. Williamson County is directly north of Travis County and includes a mix of wealthy Austin suburbs and rural areas. Its 570,431 residents are predominantly white, with fewer than 25% identifying as Hispanic or Latino. El Paso County is the western-most county in Texas. It includes both the City of El Paso and large rural areas. El Paso County is a majority-minority county, with 87.2% of its 836,915 residents identifying as Hispanic or Latino in the 2020 census.

Ethics approval for this study was obtained from the Institutional Review Board (IRB) at The University of Texas at Austin. Collected data included protected health information, and the IRB issued a waiver of informed consent. After obtaining ethics approval and appropriate data agreements with providers, we submitted structured data requests to each EMS service. Data requests were developed following consultation with key subject matter experts (SMEs) and community advisory boards. These requests identified ideal target search parameters that might indicate an overdose event within a specified timeframe of 2019–2021. The specific search parameters included targeted provider impressions such as altered mental status, confusion, withdrawal, or suicidal ideation and in-field interventions such as mechanical CPR, video laryngoscopy, or naloxone administration. Target search parameters were intentionally broad so that the acquired dataset would be likely to include a wide range of overdose and non-overdose events. A complete list of target search parameters is available in S1 Appendix. EMS provider EHR systems use different data structures and formats based on EHR brand and local implementation. Therefore, our data requests also included a data dictionary and data template to help ensure standardization across providers. Nevertheless, not all providers used all fields requested. This study used a subset of variables from the parent dataset in efforts to reduce computational power requirements when analyzing labeled data. The subset of variables includes, (a) call nature, (b) primary impression, (c) chief complaint, (d) chief narrative, (e) medication list, and (f) medical history. The definitions for these variables are found in Table 1. These six variables were chosen based on suggestions from the SMEs, who described them as the minimum amount of information needed to classify an encounter as an overdose.

**Table 1:** Key variable names and definitions.

Variable Name	Definition
**Call Nature**	A brief statement created by 9-1-1 dispatch that describes the symptom, problem, diagnosis, or other reason for the patient encounter.
**Primary Impression**	Primary medical diagnosis provided by Situation Provider (Fire Department or EMS on scene)—broad diagnosis of a patient.
**Chief Complaint**	A brief statement that describes the symptom, problem, diagnosis, or other reason for the patient encounter. The complaint is usually stated in the patient’s own words at time of incident.
**Chief Narrative**	Summary of the entire encounter reflecting procedures performed, patient outcome, impressions, etc. This is a free response summary of the encounter by first responder staff. Generally completed after an encounter is complete.
**Medication List**	An inquiry into the patient’s medical history regarding medications the patient is taking or may have recently stopped taking.
**Medical History**	It is information gained by a physician by asking specific questions, either of the patient or of other people who know the person and can give suitable information, with the aim of obtaining information useful in formulating a diagnosis and providing medical care to the patient. The medically relevant complaints reported by the patient or others familiar with the patient are referred to as symptoms, in contrast with clinical signs, which are ascertained by direct examination on the part of medical personnel. Most health encounters will result in some form of history being taken. Medical histories vary in their depth and focus.

### Ground truth annotation

In order to address significant class imbalances in the collected data, ground truth annotation for this project proceeded in two phases: (1) an initial annotation phase where one harm reduction paramedic and two supporting researchers annotated 1500 encounter notes, and (2) a second phase where provisional models developed from the first annotation set were used to oversample opioid overdose events and an additional 1458 records were annotated by two harm reduction paramedics. During the first phase, a sample of 1500 EMS encounter records were labeled based on whether the records had evidence that indicated the event was likely an opioid-related overdose. Annotations were provided by a three-person team consisting of an expert annotator and two supporting annotators who were trained to reliably replicate expert annotations. The expert annotator had been a serving EMT since 2008 and a paramedic since 2010. From 2016 through the time of the annotation, he was working as a lead outreach paramedic focusing on harm reduction in Williamson County. The expert annotator first assigned labels to a random sample of 170 records from the county where he served. A sample size of 170 records was selected because it is sufficient to achieve 90% assurance that the 95% confidence interval is no larger than 0.10 for the intraclass correlation coefficient (ICC) point estimates equal to or greater than 0.70 [23]. To assign each label, the annotator reviewed the full EHR record making note of initial on-scene impressions, history of present illness, narrative reports, and interventions performed. The supporting researchers annotated the same EHR records based on the more limited dataset that would be used to train the ML system.

Since a goal of this study was to identify possible opioid overdoses that were not easily found in structured data, the annotators used a variety of strategies to inform the annotation process. Salient information is distributed across multiple fields. In most cases, evidence of an opioid-related overdose event in one field would be insufficient to assign the opioid overdose code. Annotators would, therefore, synthesize information across fields in order to assess when events were likely opioid-related overdoses. For some encounters, patients would disclose to providers what substances were ingested by name or type (i.e., opioid, antidepressant, alcohol), and this disclosure would be documented in the chief narrative and/or chief complaint fields. This is a strong indicator of an opioid-related overdose. When the patient was unconscious, not forthcoming about possible drug use, or the providers were unable to collect relevant information from bystanders or family, the annotators had to use more context clues to determine the nature of the event. For some encounters, EMS providers would document in the impressions or chief narrative that they suspected an opioid overdose despite the lack of confirmation from patients or bystanders. Additionally, if naloxone was administered, this would imply that the paramedics suspected opioid involvement. Naloxone may be administered in the field as a precaution if a patient is experiencing respiratory depression and an EMS practitioner suspects possible opioid overdose; however, naloxone administration alone is insufficient to identify an opioid overdose absent other context clues. Treatment narratives typically also document if the naloxone had an effect on the patient, such as increasing responsiveness which signified the naloxone was successful. This provides supporting evidence that an opioid was the cause of their symptoms. Common symptoms and signs of opioid overdose present in chief narratives or chief complaint fields were also used to inform annotations. Examples of key symptoms include pinpoint pupils, shortness of breath, decreased cognition, altered mental status, decreased respiratory rate, vomiting, choking sounds, blue skin, snoring sounds, and unresponsiveness. Interrater reliability was assessed on the sample of 170 records using ICC [24]. The supporting annotators achieved reliability scores of ICC = 0.713 and ICC = 0.813 when compared to the expert rater with full access to the EHR records. These scores indicate moderate and good reliability, respectively. Once sufficient reliability had been achieved, both the expert and supporting annotators independently labeled the remaining records for the phase one testing and training data sets.

Feature engineering and modeling techniques (described below) were applied to the training sets and the highest preforming model was used to classify all available data. A random sample of 100 predicted opioid overdose events and 100 predicted non-opioid overdose events was prepared for gold standard annotation by two harm reduction paramedics. The first paramedic was the same as the expert annotator in phase one. Predicted classes were removed, the order was randomized, and each expert annotated all 200 cases. Phase two inter-rater reliability was ICC = 0.922. The two raters proceeded independently on randomly selected and randomized 200-item batches. As mentioned previously, this two-phase approach allowed us to partially remediate class imbalances and develop a more performant system using a smaller dataset. Classifier performance was interactively tested as new annotation sets were completed until at least one model returned an AUROC 95% confidence interval with a low end of achieved an AUROC ≥ 0.90.

Annotations from phases one and two were combined into a final training and test set of 2958 cases. (A small number of duplicates were included in both phases in order to evaluate annotation drift.) The final dataset used in this study included 1,635 records from Travis County, 747 records from El Paso County, and 576 records from Williamson County. The annotation team identified a total of 438 opioid-related overdose events in the collected records. There were 99 overdoses in El Paso County, 289 in Travis County, and 50 in Williamson County. The largest share of overdoses was among patients identified as White by EMS (N = 241, 55.0%). There were 242 (25.8%) and 24 (5.5%) identified overdose events among Hispanic or Latino and Black or African American patients respectively. There was a single overdose event for an Asian identified person (1.4%), and 59 of the identified overdoses (13.5%) were for patients with unknown or unassigned ethnicity. A majority of overdose patients were male (N = 315, 71.9%), and the remaining were female (N = 123, 28.1%). Overdose patients ranged in age from 16 to 90 with an average reported or estimated age of 38.28. Patient demographics rely on reported data from emergency services. Data provided did not disaggregate race and ethnicity or identify any patients as being non-binary or transgender. Complete demographic details are available in the S2 Appendix.

### Feature engineering

In each of the two phases, we implemented three different approaches to feature engineering: (1) TF-IDF, (2) Cui2Vec concept embeddings, and (3) an empirically-derived custom “flags” approach. TF-IDF is the product of the term frequency (in a document) and the term’s inverse document frequency (across the training or test set). This statistic is useful for identifying salient terms for text classification. Vectorization techniques are among the most popular contemporary approaches to machine learning. They leverage language models trained on large textual datasets to distill text data into mathematical representations of word meanings. Vectorization approaches are popular because they can be highly performant out-of-the-box for a wide range of datasets. For the purposes of this study, we used the ClinSpacy framework [25]. ClinSpacy is the R implementation of the Cui2Vec concept model [22]. This model provides concept vectors for common unique concepts identified in the Unified Medical Language System (UMLS). Cui2Vec is one of the largest collections of medical concepts (108,477) available and was trained on a dataset of 20 million clinical notes and 1.7 million full text journal articles. Although Cui2Vec is no longer state-of-the-art, it remains highly preformat, exceeding benchmarks for several popular transformers-based models, including BERT-L and BlueBERT [26]. Despite high performance on benchmarks, vectorization based on large concepts models often results in large feature sets, which can lead to overfitting, where a model effectively memorizes the training data [27]. Vectorization of unstructured clinical narratives has been shown to lead to overfitting in various use cases [28]. Within this study, vectorization of all selected EHR fields would be likely to lead to similar model overfitting. Therefore, our approach involved extracting the Cui2Vec vector embeddings for the chief narratives exclusively in order to reduce the risks of overfitting. Additionally, since the chief narrative is intended to be a complete summary of the encounter, it is the ideal single field on which to base a classification.

Custom feature engineering based on qualitative analyses of textual data has previously been shown to be capable of producing ML models that meet or exceed the performance level of more computationally expensive approaches [29]. In essence, custom feature engineering involves the inductive identification of keywords and “flagging” their presence in the data. The custom flag approach is not only capable of producing performant models, it also is far less computationally expensive than vectorization techniques. Additionally, the custom flags approach is highly interpretable. Users not familiar with vectorization or similar, less intuitive feature engineering approaches can readily understand keyword frequencies as predictive of classifications. In this study, our approach involved identifying keywords for opioids and overdoses. Keywords were identified through an integrative review of qualitative interview transcripts with community informants and subsequent comparison to the language typical of EMS records. Final keyword lists are available in Table 2. Selected keywords overlap substantially with, but are not identical to, the opioid and overdose keywords used by the CDC’s DOSE System. DOSE makes primary assessments based on ICD-10 and similar diagnostic codes, but supports case identification with opioid and overdose flags identified from chief complaint fields in ED EHRs [30]. We used a regular expressions framework to identify and count keywords across data fields. Our particular implementation was case insensitive and enforced word boundary requirements around abbreviations like “OD” in order to avoid false positives.

**Table 2:** Term sets and keywords.

Term Set	
**Opioid terms**	opioid, opiate, Narcan, naloxone, heroin, methadone, fentanyl, Percocet, oxycontin, oxycodone, Vicodin, morphine, crack, cocaine, Tylenol 3, codeine, oxy, tramadol
**Overdose terms**	ingestion, substance, abuse, intox, poisoning, OD, overdose

### Modeling, validation, and explainability

Candidate feature sets were tested with multiple models and ensemble options. Specifically, we trained models for each feature set using a Generalized Linear Model (GLM), a single-hidden-layer feed-forward neural network, Naive Bayes, and the Extreme Gradient Boost (XGBoost) algorithm. All models were trained and ensembled using the linear modeling approach in CaretEnsemble [31]. Models were trained with random search hyperparameter optimization and using k-fold cross-validation (k = 5 with 10 repeats) to further reduce the risk of overfitting. Hyperparameter optimization was assessed with area under the receiver operating characteristic curve (AUROC), and final benchmarking was conducted using an 80/20 test/train split. For each feature engineering and model combination, we calculated AUROC to measure overall performance and the 95% confidence interval using DeLong’s method [32] as implemented by Sun and Xu [33]. Finally, we also extracted data on the relative importance of each variable used in the custom features models.

## Results

**Fig 1:**
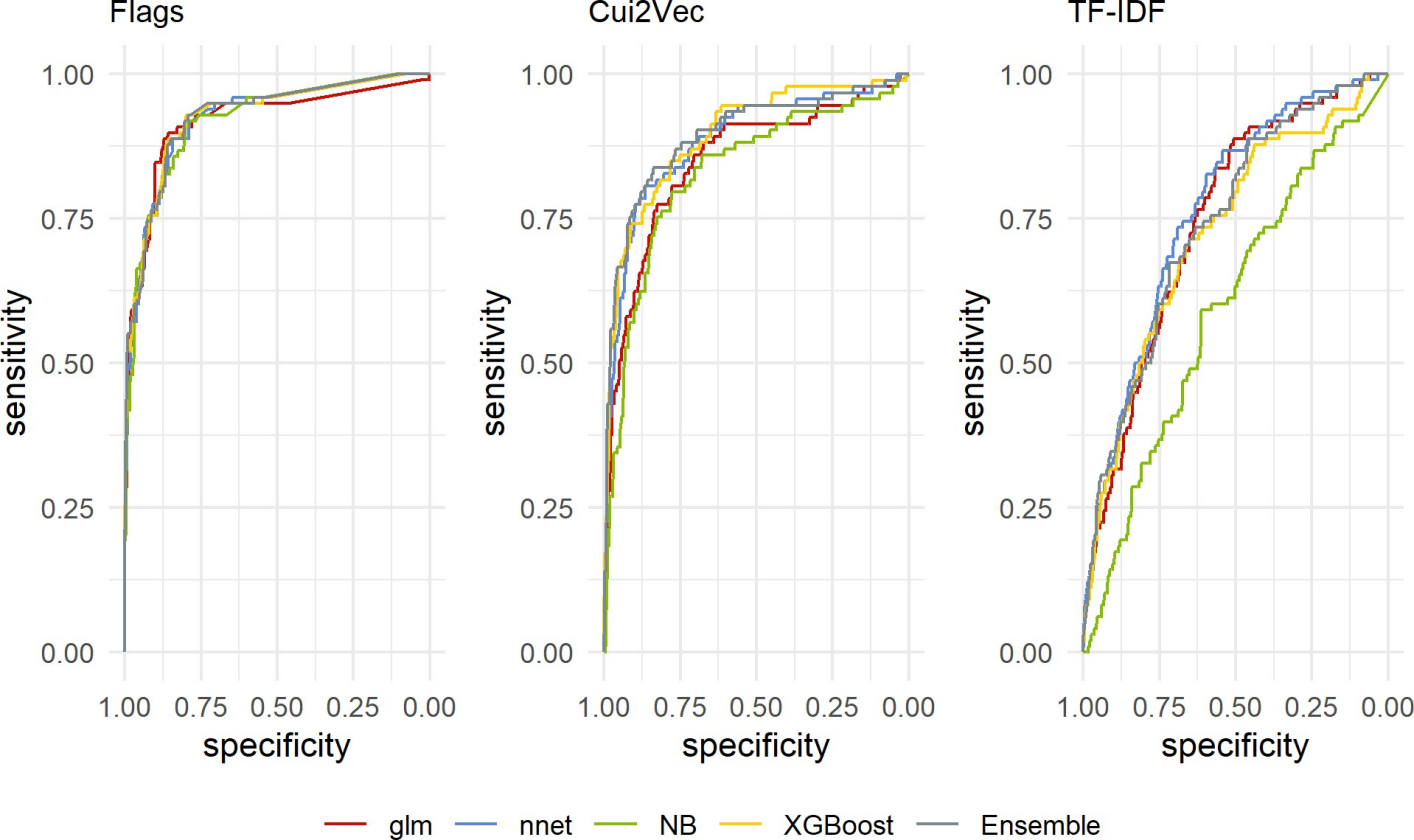
ROC curves for candidate feature sets and models.

In each phase, we trained twelve models and assembled three ensembles to identify opioid overdoses in EMS encounter data. Phase one models trained using TF-IDF ranged from AUROC = 0.39 for the GLM to 0.74 for the neural network. Models trained using Cui2Vec features ranged from AUROC = 0.364 for the GLM to AUROC = 0.81 for the neural net and the ensemble. Finally, models trained using custom features were generally more performant, with benchmarks from AUROC = 0.78 with the Naive Bayes model to 0.85 for the four-model ensemble. The full range of phase one AUROC scores and 95% confidence intervals are available in Table 3. Phase two models trained using TF-IDF feature engineering ranged from AUROC = 0.59 (95% CI: 0.53–0.66) for the Naïve Bayes to AUROC = 0.76 (95% CI: 0.71–0.81) for the neural network. Models trained using concept vectorization features ranged from AUROC = 0.83 (95% 0.78–0.88)for the Naïve Bayes to AUROC = 0.89 (95% CI: 0.85–0.94) for the ensemble. Models trained using custom features were the most performant, with benchmarks ranging from AUROC = 0.92 (95% CI: 0.88–0.95) with the GLM to 0.93 (95% CI: 0.90–0.96)for the ensemble. Complete Phase Two AUROC results are available in Table 4 and phase two ROC curves for each model and ensemble are plotted in Fig 1. Ultimately, these data indicate that the lightweight and interpretable flags approach consistently creates more performant models than either the TF-IDF or Cui2Vec embeddings approaches.

**Table 3:** Phase one AUROC scores and 95% CIs for candidate feature sets and models.

	Flags	TF-IDF	Cui2Vec
**GLM**	0.80 (0.70–0.89)	0.39 (0.29–0.50)	0.36 (0.27–0.46)
**NNet**	0.84 (0.75–0.93)	0.74 (0.63–0.86)	0.81 (0.70–0.92)
**Naïve Bayes**	0.78 (0.67–0.88)	0.52 (0.51–0.53)	0.71 (0.59–0.83)
**xgbTree**	0.84 (0.76–0.91)	0.68 (0.57–0.80)	0.79 (0.67–0.91)
**Ensemble**	0.85 (0.77–0.93)	0.73 (0.61–0.85)	0.81 (0.70–0.92)

**Table 4:** Phase two AUROC scores and 95% CIs for candidate feature sets and models.

	Flags	TF-IDF	Cui2Vec
**GLM**	0.92 (0.88–0.95)	0.74 (0.69–0.79)	0.85 (0.80–0.90)
**NNet**	0.92 (0.89–0.95)	0.76 (0.71–0.81)	0.88 (0.84–0.93)
**Naïve Bayes**	0.92 (0.89–0.95)	0.59 (0.53–0.65)	0.83 (0.78–0.88)
**xgbTree**	0.93 (0.89–0.96)	0.72 (0.67–0.78)	0.89 (0.86–0.93)
**Ensemble**	0.93 (0.90–0.96)	0.74 (0.69–0.79)	0.89 (0.85–0.94)

The phase two flags ensemble was the most performant opioid overdose classifier at AUROC = 0.93. Performance and error analyses were based on the application of the model to a reserved testing set of 592 EMS encounters. Table 5 provides the confusion matrix showing 486 true negatives, 45 true positives, 44 false negatives, and 8 false positives overall. We conducted two targeted demographics-based error analyses designed to identify potential disparities by ethnicity or gender. Error rates range from a <1% false positive rate among patients without an identified or assigned ethnicity to a 8.99% false negative rate among White patients. See S2 Appendix for complete details. Pairwise equality of proportions tests indicates that there is no significant difference among error rates by ethnicity. Error rates by gender ranged from 1.12% for female false positives to 11.4% for male false negatives. See S2 Appendix for complete details. A pairwise equality of proportions test found a significant differences in false negative rates by gender χ^2^ = 13.70, p = 0.0002. No significant difference was observed for false positives by gender.

**Table 5:** Confusion matrix for the phase two flags ensemble model.

		Reference	
		Non-OOD	OOD
**Prediction**	**Non-OOD**	486	44
	**OOD**	8	54

We also conducted a manual error analysis on each prediction error (N = 52). Among the 44 false negatives, the most common sources of error were positive Narcan response (N = 25), misleading chief complaint (N = 8), report of unknown drug product use (N = 6), narratives focusing on scene descriptions, and narratives focusing on suicidal ideation and/or attempts (N = 3). A key indicator of opioid overdose for the annotation team was a positive response to Narcan administration. However, because Narcan is frequently administered in cases where the cause of altered mental status or loss of consciousness is suspected to be (but not actually) resultant from opioid overdose, Narcan administration (without subsequent improvement) is an unreliable predictor of overdose. The flag approach to feature engineering cannot relate the Narcan keyword to subsequent outcomes in the way a human annotator can. The second most leading likely sources of error is misleading chief complaints. In some cases, patients or bystanders complain of certain symptoms that may not indicate an opioid OD, but subsequent clinical investigation reveals a fuller picture. In several cases the chief complaint was related to a fall event, and EMS personnel later determined that the fall was caused by altered mental status due to opioid OD. In several cases the keyword flagging approach did not pick up key indicators for opioid use that come from unclearly identified products, e.g., “unknown pain pills” or “blue pills.” Additionally, EMS personnel frequently encounter dynamic, unpredictable, and dangerous situations. In these cases, the bulk of the chief narrative tends to describe relevant hazards and events (e.g., efforts to safely remove a patient with altered mental status from a busy freeway). This results in reduced narrative content related to diagnosis and treatment and leads to false negatives. The remaining false negatives tended to arise from attempted suicides by OD. In these cases the chief narratives focused on behavioral health markers and there were subsequently an insufficient number of opioid or OD-related flags to reach the decision threshold. Of the eight identified false positives, four were cases on non-Opioid overdoses, three were cases where the patient had a history of opioid use that was not relevant to the current case, and one case of detailed denial. That is, when patients deny taking multiple specific opioids, it increases the flag count and is more likely to trigger an opioid overdose classification.

**Fig 2:**
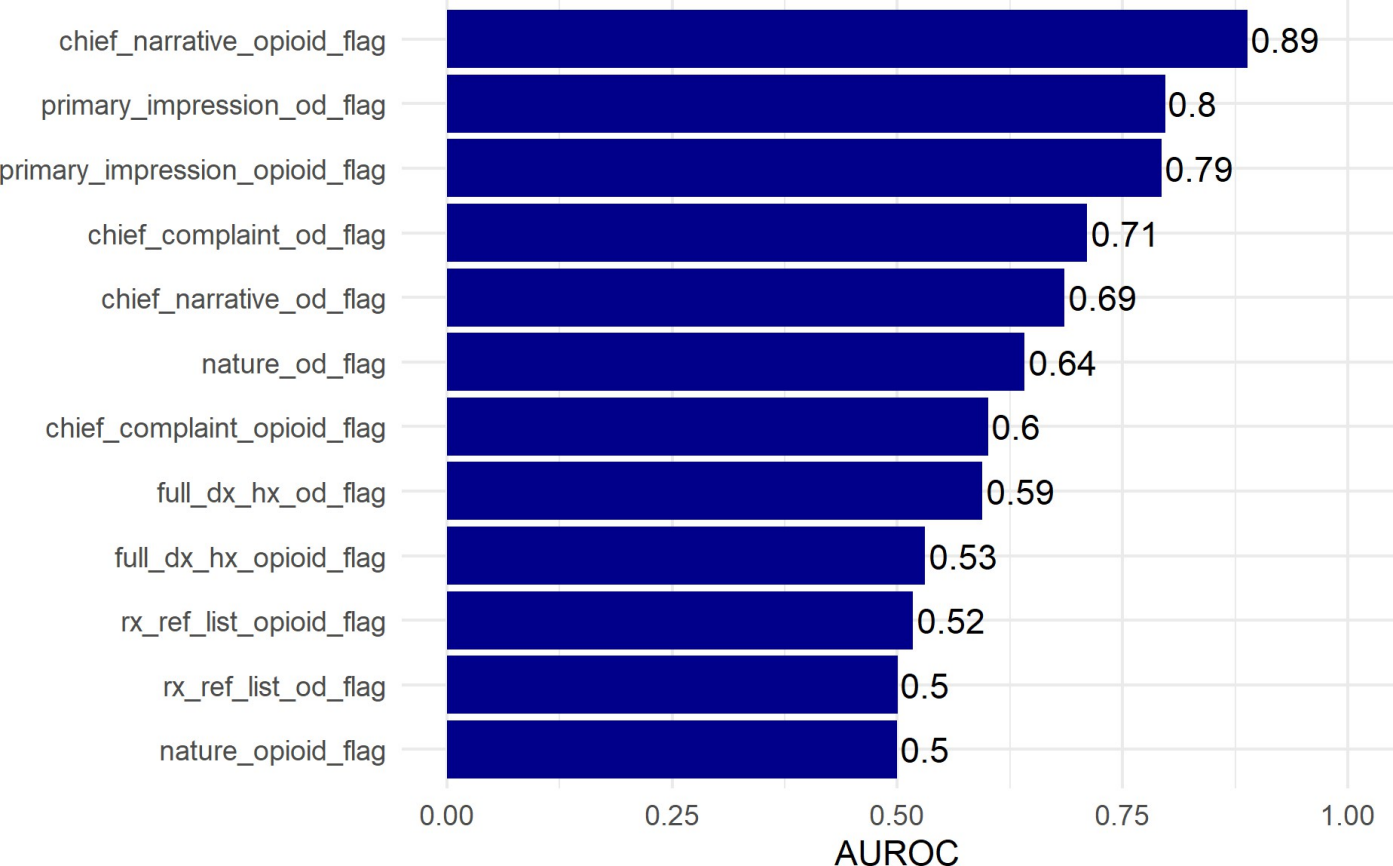
Variable importance for custom features models and ensemble.

Finally, variable importance data for each flag predictor is available in Fig 2. Model-independent importance was determined by calculating the AUROC for each predictor independently. Variable importance data indicate that opioid flags in the chief narrative and overdose flags in the primary impressions are the most important. The variable importance analysis further indicates that patient history, prescription lists and call nature tend to be less important to making reliable opioid predictions.

## Discussion

The results presented here indicate that it is possible to develop lightweight, interpretable, and performant models for opioid overdose surveillance based on unstructured text data in EMS records. Specifically, the custom flags approach, which leverages expert-guided qualitative analysis, proved to be the most performant, with an ensemble AUROC of 0.92. This notably exceeds the performance of preexisting frameworks that train data based on ICD-10 labels and/or use naloxone administration as a proxy for opioid overdose identification. Prior research in this area has found that naloxone administration to be a poor proxy for opioid-involved overdoses [34], with roughly 60% positive predictive value (PPV) for correctly diagnosing opioid overdoses [35]. Recent research has also found that using linked ICD-10 codes for labeling EMS data achieves an underwhelming 27.2% PPV, but that augmenting ICD-10 codes with overdose keywords can raise the PPV to 59.8% [14]. In contrast, our custom features approach returned PPVs ranging from 80% to 100%. Although, it should be noted that the Naïve Bayes model which achieved PPV = 100 performed poorly on specificity and thus we would not recommend its use. Ultimately, the custom features approach has the benefit of being less computationally expensive and highly interpretable. Model users and decision-makers without expertise in ML development or feature engineering can readily understand the variable importance data.

The development of efficient and performant ML systems for opioid overdose surveillance has the potential to support improved response to the opioid epidemic. Current opioid overdose surveillance regimens are driven by a focus on ED clinical records and the collection of all-cause mortality data. While these data sources are effective for many use cases, they cannot effectively support real-time public health responses or location-aware community-embedded harm reduction initiatives. Effectively addressing the opioid epidemic will require access to reliable, timely, and location-aware data on opioid overdoses [5, 7]. Centralized records like those of ED departments and medical examiners typically lack important site-of-use data and are subject to significant reporting delays. Most importantly, these surveillance systems suffer from significant reporting gaps for non-fatal overdose events and could be dramatically improved when augmented by reliable analyses of EMS encounter data. The results presented in this study provide an effective and economic framework for integrating these critically missing data.

While the proposed ML framework is promising in terms of its ability to better support opioid overdose surveillance and local harm reduction initiatives, the techniques outlined here come with several limitations. While the keyword flagging approach to feature engineering is quite performant, it does poorly in cases where opioid overdoses can be identified based on positive response to Narcan administration. Future research should explore adjunctive feature engineering techniques and/or the inclusion of additional data fields so as to address this issue. Furthermore, this kind of situation may also be an ideal use-case for large language models which show promising results for cause-effect classification [36]. Additionally, our findings add further support to growing awareness that distinguishing between overdoses and behavioral health emergencies can be difficult in EMS records [37]. Another limitation is that the approach described in this study effectively identifies opioid overdose events but does not provide additional information that would be useful to first responders and public health agencies. For example, future research in this area should explore the possibility of developing non-binary classifiers that can distinguish between mono-opioid and polysubstance overdose events. It may also be useful for future studies to develop ML approaches that can reliably differentiate between accidental and intentional overdoses. This could be especially important given the data here demonstrating that the current system may be less performant in classifying intentionally overdoes. Finally, future research in this area should train models using larger datasets drawn from an increasing diversity of EMS contexts. EMS documentation can vary quite widely based on state and local laws, different service types (paramedic vs. fire and rescue), and different electronic health records providers. For automatic opioid overdose classifiers to be effective across use-cases, data sets would need to be drawn from a wide range of providers. Furthermore, due to the relative sparsity of opioid overdose events (even in datasets pre-filtered by impression), larger samples will be required to develop performant classifiers that can reliably distinguish among different event types. And finally, significant bodies of research demonstrate that health sector ML can produce biased outcomes when systems trained on data from one population are applied to other populations [38–40]. While the models developed in this study were trained using data from multiple contexts and locations, the specific techniques discussed here may not be as performant for other state and county contexts. Given these challenges, the most effective short-term approach would likely be one based in localization. That is, individual services might consider using the techniques described here to develop models optimized for their particular data management and demographic contexts.

## Conclusion

This article reports on our efforts to create a lightweight and interpretable binary classifier that identifies opioid overdose events in county EMS records. We compared TF-IDF and Cui2Vec to a lightweight and interpretable custom features alternative. All feature engineering approaches were trained using a range of model architectures and linear model ensembling. Each approach produced performant classifiers that could be used to effectively support improved opioid overdose surveillance. However, the custom features approach has the benefit of being computationally inexpensive and highly interpretable. Future research in this area should extend these efforts by developing non-binary classifiers that can distinguish among multiple overdose event types. Nevertheless, the current system can effectively enhance opioid overdose surveillance, augmenting already available all-cause mortality data and the CDC DOSE statistics. Most importantly, data and subsequent analysis resulting from the application of this ML system to county EMS records can help better inform both county public health and community harm reduction organization responses to the opioid epidemic. There is particular promise for leveraging location aware EMS datasets to more effectively allocate resources for harm reduction, and the methodological approach described here can be deployed to produce near real-time data in support of more timely public health interventions.

## Supporting information

S1 Appendix Filters used by first responder data sources. (DOCX)

S2 Appendix EMS encounters, opioid overdose events, and errors by reported or assigned patient ethnicity and gender. (DOCX)

## References

[1] Provisional Data Shows U.S. Drug Overdose Deaths Top 100,000 in 2022 | Blogs | CDC [Internet]. 2023 [cited 2023 Jun 19]. Available from: https://blogs.cdc.gov/nchs/2023/05/18/7365/

[2] Langabeer JR, Stotts AL, Bobrow BJ, Wang HE, Chambers KA, Yatsco AJ, et al. Prevalence and charges of opioid-related visits to U.S. emergency departments. Drug Alcohol Depend. 2021 Apr 1;221:108568. doi: 10.1016/j.drugalcdep.2021.108568 33578297

[3] Soares WE, Melnick ER, Nath B, D’Onofrio G, Paek H, Skains RM, et al. Emergency Department Visits for Nonfatal Opioid Overdose During the COVID-19 Pandemic Across Six US Health Care Systems. Ann Emerg Med. 2022 Feb 1;79(2):158–67. doi: 10.1016/j.annemergmed.2021.03.013 34119326 PMC8449788

[4] Buben A, Cance JD. Uncertainty in overdose death reporting impedes the public health response. Am J Drug Alcohol Abuse. 2021 Nov 2;47(6):655–7. doi: 10.1080/00952990.2021.1977312 34606400

[5] Claborn K, Creech S, Conway FN, Clinton NM, Brinkley KT, Lippard E, et al. Development of a digital platform to improve community response to overdose and prevention among harm reduction organizations. Harm Reduct J. 2022 Jun 3;19(1):62. doi: 10.1186/s12954-022-00636-2 35658871 PMC9164184

[6] Jalali MS, Ewing E, Bannister CB, Glos L, Eggers S, Lim TY, et al. Data Needs in Opioid Systems Modeling: Challenges and Future Directions. Am J Prev Med. 2021 Feb 1;60(2):e95–105. doi: 10.1016/j.amepre.2020.08.017 33272714 PMC8061725

[7] Gupta R, Holtgrave DR. A National Tracking System for Nonfatal Drug Overdoses. JAMA [Internet]. 2022 Jun 30 [cited 2022 Jul 8]; Available from: 10.1001/jama.2022.10815 35771586

[8] Rosenbaum JE, Stillo M, Graves N, Rivera R. Timeliness of provisional United States mortality data releases during the COVID-19 pandemic: delays associated with electronic death registration system and weekly mortality. J Public Health Policy. 2021;42(4):536–49. doi: 10.1057/s41271-021-00309-7 34732841 PMC8564267

[9] Rock P, Singleton M. EMS Heroin Overdoses with Refusal to Transport & Impacts on ED Overdose Surveillance. Online J Public Health Inform. 2019 May 30;11(1):e430.

[10] Rowe CL, Santos GM, Kornbluh W, Bhardwaj S, Faul M, Coffin PO. Using ICD-10-CM codes to detect illicit substance use: A comparison with retrospective self-report. Drug Alcohol Depend. 2021 Apr 1;221:108537. doi: 10.1016/j.drugalcdep.2021.108537 33621806 PMC11008535

[11] Ward PJ, Rock PJ, Slavova S, Young AM, Bunn TL, Kavuluru R. Enhancing timeliness of drug overdose mortality surveillance: A machine learning approach. PLOS ONE. 2019 Oct 16;14(10):e0223318. doi: 10.1371/journal.pone.0223318 31618226 PMC6795484

[12] Neill DB, Herlands W. Machine Learning for Drug Overdose Surveillance. J Technol Hum Serv. 2018 Jan 2;36(1):8–14.

[13] Campo DS, Gussler JW, Sue A, Skums P, Khudyakov Y. Accurate spatiotemporal mapping of drug overdose deaths by machine learning of drug-related web-searches. PLOS ONE. 2020 Dec 7;15(12):e0243622. doi: 10.1371/journal.pone.0243622 33284864 PMC7721465

[14] Fix J, Ising AI, Proescholdbell SK, Falls DM, Wolff CS, Fernandez AR, et al. Linking Emergency Medical Services and Emergency Department Data to Improve Overdose Surveillance in North Carolina. Public Health Rep. 2021 Nov 1;136(1_suppl):54S–61S. doi: 10.1177/00333549211012400 34726971 PMC8573781

[15] Bozorgi P, Porter DE, Eberth JM, Eidson JP, Karami A. The leading neighborhood-level predictors of drug overdose: A mixed machine learning and spatial approach. Drug Alcohol Depend. 2021 Dec 1;229:109143. doi: 10.1016/j.drugalcdep.2021.109143 34794060

[16] Volkow ND, Chandler RK, Villani J. Need for comprehensive and timely data to address the opioid overdose epidemic without a blindfold. Addict Abingdon Engl. 2022 Aug;117(8):2132–4. doi: 10.1111/add.15957 35611646

[17] Crosier B, Borodovsky J, Mateu-Gelabert P, Guarino H. Finding a needle in the haystack: Using machine-learning to predict overdose in opioid users. Drug Alcohol Depend. 2017 Feb 1;171:e49.

[18] Radloff CL, Bennett H, Staes CJ. Utility of Poison Control Center Data for Automated Opioid Overdose Surveillance. J Public Health Manag Pract. 2022 Jun;28(3):272. doi: 10.1097/PHH.0000000000001494 35334484

[19] Sarker A, Gonzalez-Hernandez G, Ruan Y, Perrone J. Machine Learning and Natural Language Processing for Geolocation-Centric Monitoring and Characterization of Opioid-Related Social Media Chatter. JAMA Netw Open. 2019 Nov 6;2(11):e1914672. doi: 10.1001/jamanetworkopen.2019.14672 31693125 PMC6865282

[20] Prieto JT, Scott K, McEwen D, Podewils LJ, Al-Tayyib A, Robinson J, et al. The Detection of Opioid Misuse and Heroin Use From Paramedic Response Documentation: Machine Learning for Improved Surveillance. J Med Internet Res. 2020 Jan 3;22(1):e15645. doi: 10.2196/15645 31899451 PMC6969388

[21] Khare A, Sidana A, Mohemmed A, Allicock DM, Waterstone A, Zimmer MA, et al. Acceleration of opioid-related EMS runs in the spring of 2020: The National Emergency Medical Services Information System data for 2018–2020. Drug Alcohol Depend. 2022 Mar 1;232:109271. doi: 10.1016/j.drugalcdep.2022.109271 35051696

[22] Beam AL, Kompa B, Schmaltz A, Fried I, Weber G, Palmer NP, et al. Clinical Concept Embeddings Learned from Massive Sources of Multimodal Medical Data [Internet]. arXiv; 2019 [cited 2022 Jun 16]. Available from: http://arxiv.org/abs/1804.01486 PMC6922053 31797605

[23] Zou GY. Sample size formulas for estimating intraclass correlation coefficients with precision and assurance. Stat Med. 2012;31(29):3972–81. doi: 10.1002/sim.5466 22764084

[24] Bartko JJ. The Intraclass Correlation Coefficient as a Measure of Reliability. Psychol Rep. 1966 Aug 1;19(1):3–11. doi: 10.2466/pr0.1966.19.1.3 5942109

[25] Singh K, Kompa B, Beam A, Schmaltz A. clinspacy: Clinical Natural Language Processing using “spaCy”, “scispaCy”, and “medspaCy” [Internet]. 2021 [cited 2022 Mar 3]. Available from: https://CRAN.R-project.org/package=clinspacy

[26] Mao Y, Fung KW. Use of word and graph embedding to measure semantic relatedness between Unified Medical Language System concepts. J Am Med Inform Assoc. 2020 Oct 1;27(10):1538–46. doi: 10.1093/jamia/ocaa136 33029614 PMC7566472

[27] Abdul Salam M, Taher A, Samy M, Mohamed K. The Effect of Different Dimensionality Reduction Techniques on Machine Learning Overfitting Problem. Int J Adv Comput Sci Appl. 2021 Jan 1;12.

[28] Zhan X, Humbert-Droz M, Mukherjee P, Gevaert O. Structuring clinical text with AI: Old versus new natural language processing techniques evaluated on eight common cardiovascular diseases. Patterns. 2021 Jul 9;2(7):100289. doi: 10.1016/j.patter.2021.100289 34286303 PMC8276012

[29] Majdik ZP, Wynn J. Building Better Machine Learning Models for Rhetorical Analyses: The Use of Rhetorical Feature Sets for Training Artificial Neural Network Models. Tech Commun Q. 2022 May 13;0(0):1–16.

[30] CDC’s Drug Overdose Surveillance and Epidemiology (DOSE) System | Drug Overdose | CDC Injury Center [Internet]. 2022 [cited 2022 Jun 16]. Available from: https://www.cdc.gov/drugoverdose/nonfatal/case.html

[31] Deane-Mayer ZA, Knowles JE. caretEnsemble: Ensembles of Caret Models. 2019. R Package Version. 2(1):35.

[32] DeLong ER, DeLong DM, Clarke-Pearson DL. Comparing the Areas under Two or More Correlated Receiver Operating Characteristic Curves: A Nonparametric Approach. Biometrics. 1988;44(3):837–45. 3203132

[33] Sun X, Xu W. Fast Implementation of DeLong’s Algorithm for Comparing the Areas Under Correlated Receiver Operating Characteristic Curves. IEEE Signal Process Lett. 2014 Nov;21(11):1389–93.

[34] Chris Smith J, Burr WS. Ineffectiveness of Paramedic Naloxone Administration as a Standalone Metric for Community Opioid Overdoses and the Increasing Use of Naloxone by Community Members. Prehosp Emerg Care. 2023 Apr 3;27(3):328–33. doi: 10.1080/10903127.2022.2033895 35073227

[35] Grover JM, Alabdrabalnabi T, Patel MD, Bachman MW, Platts-Mills TF, Cabanas JG, et al. Measuring a Crisis: Questioning the Use of Naloxone Administrations as a Marker for Opioid Overdoses in a Large U.S. EMS System. Prehosp Emerg Care. 2018 Jun;22(3):281–9. doi: 10.1080/10903127.2017.1387628 29297739

[36] Hosseini P, Broniatowski DA, Diab M. Knowledge-Augmented Language Models for Cause-Effect Relation Classification. In: Proceedings of the First Workshop on Commonsense Representation and Reasoning (CSRR 2022) [Internet]. Dublin, Ireland: Association for Computational Linguistics; 2022 [cited 2023 Jun 19]. p. 43–8. Available from: https://aclanthology.org/2022.csrr-1.6

[37] Rivard MK, Cash RE, Chrzan K, Powell J, Kaye G, Salsberry P, et al. Public Health Surveillance of Behavioral Health Emergencies through Emergency Medical Services Data. Prehosp Emerg Care. 2022 Nov 2;26(6):792–800. doi: 10.1080/10903127.2021.1973626 34469269

[38] McCradden MD, Joshi S, Anderson JA, Mazwi M, Goldenberg A, Zlotnik Shaul R. Patient safety and quality improvement: Ethical principles for a regulatory approach to bias in healthcare machine learning. J Am Med Inform Assoc. 2020 Dec 9;27(12):2024–7. doi: 10.1093/jamia/ocaa085 32585698 PMC7727331

[39] Obermeyer Z, Powers B, Vogeli C, Mullainathan S. Dissecting racial bias in an algorithm used to manage the health of populations. Science. 2019 Oct 25;366(6464):447–53. doi: 10.1126/science.aax2342 31649194

[40] Parikh RB, Teeple S, Navathe AS. Addressing Bias in Artificial Intelligence in Health Care. JAMA. 2019 Dec 24;322(24):2377–8. doi: 10.1001/jama.2019.18058 31755905

