# Supplementary material for: An interpretable machine learning framework for opioid overdose surveillance from emergency medical services records
Source: PLoS One. 2024 Jan 30;19(1):e0292170. doi: 10.1371/journal.pone.0292170 (PMC10826931; doi:10.1371/journal.pone.0292170)
Supplement: S1 Appendix — (DOCX) [file pone.0292170.s001.docx]

**An interpretable machine learning framework for opioid overdose surveillance from emergency medical services records**

**S1 Appendix****:** *Filters used by first responder data sources*

| **Impression Filters** | | |
| --- | --- | --- |
| Alcohol dependence with withdrawal | Altered Mental Status | Anxiety reaction/Emotional upset |
| Behavioral/psychiatric episode | Cannabis related disorder | Cocaine related disorders |
| Confusion/Delirium | Hallucinogen related disorders | Inhalant related disorders |
| Opioid related disorders | Other stimulant related disorders | Overdose - Acetaminophen |
| Overdose - Alcohol | Overdose - Amphetamine | Overdose - Benzodiazepine |
| Overdose - Cannabis | Overdose - Cocaine | Overdose - Hallucinogens |
| Overdose - Heroin | Overdose - Methadone | Overdose - Opium |
| Overdose - Other opioids | Overdose - Synthetic marijuana | Overdose - Synthetic narcotics |
| Overdose - Unspecified | Respiratory Arrest | Respiratory Failure |
| Sedative, hypnotic, or anxiolytic related disorders | Seizures without status epilepticus | Suicidal Ideation |
| Suicide attempt |  |  |
| **Treatment Filters** | | |
| CPR | Epinephrine 1:10 | Epinephrine Infusion |
| Ketamine | Manual Defibrillation | Mechanical CPR |
| Midazolam | Naloxone | Narcan |
| Nasotracheal Intubation | Rapid Sequence Intubation (RSI) | Rocuronium |
| Suction | Video Laryngoscopy | Zofran |
