# Supplementary material for: An interpretable machine learning framework for opioid overdose surveillance from emergency medical services records
Source: PLoS One. 2024 Jan 30;19(1):e0292170. doi: 10.1371/journal.pone.0292170 (PMC10826931; doi:10.1371/journal.pone.0292170)
Supplement: S2 Appendix — (DOCX) [file pone.0292170.s002.docx]

**An interpretable machine learning framework for opioid overdose surveillance from emergency medical services records**

**S2 Appendix:** *EMS Encounters, Opioid Overdose Events, and Errors by Reported or Assigned Patient Ethnicity and Gender*

*EMS Encounters and Opioid Overdose Events by Reported or Assigned Patient Ethnicity*

| Reported or Assigned Ethnicity | Total Encounters | Opioid Overdose Events (N) | Opioid Overdose Events (%) |
| --- | --- | --- | --- |
| American Indian or Alaska Native | 1 | 0 | 0 |
| Asian | 23 | 1 | 1.37 |
| Black or African American | 319 | 24 | 5.48 |
| Hispanic or Latino | 683 | 113 | 25.8 |
| Native Hawaiian or Other Pacific Islander | 2 | 0 | 0 |
| White | 1361 | 241 | 55.0 |
| Uknown or Unassigned | 413 | 59 | 13..47 |

*EMS Encounters and Opioid Overdose Events by Reported or Assigned Patient Gender*

| Gender | Total Encounters | Opioid Overdose Events (N) | Opioid Overdose Events (%) |
| --- | --- | --- | --- |
| Female | 1345 | 123 | 28.1 |
| Male | 1610 | 315 | 71.9 |
| Unknown | 3 | 0 | 0 |

*Errors by Reported or Assigned Ethnicity*

|  | False Negatives | | False Positives | |
| --- | --- | --- | --- | --- |
| Reported or Assigned Ethnicity | *N* | *Percent* | *N* | *Percent* |
| *American Indian or Alaska Native* | 0 | 0 | 0 | 0 |
| *Asian* | 0 | 0 | 0 | 0 |
| *Black or African American* | 3 | 5.26 | 0 | 0 |
| *Hispanic or Latino* | 9 | 6.47 | 5 | 3.6 |
| *White* | 24 | 8.99 | 2 | 0.74 |
| *Uknown or Unassigned* | 8 | 7.41 | 1 | 0.93 |

*Errors by Reported or Assigned Gender*

|  | False Negatives | | False Positives | |
| --- | --- | --- | --- | --- |
| Reported or Assigned Gender | *N* | *Percent* | *N* | *Percent* |
| *Female* | 8 | 2.97 | 3 | 1.12 |
| *Male* | 36 | 11.4 | 5 | 1.15 |
